# YouBelong Home: A Ugandan Community Mental Health Intervention

**DOI:** 10.1007/s10597-022-01058-x

**Published:** 2022-12-07

**Authors:** D. Cappo, B. Mutamba, K. Ayesiga, E. Kebirungi, D. Chelangat, G. Fegan, S.T. Jacob, E. Nsangi, I. Ntabazi, D. Nalubwama, N. Nakasujja, E. Odoki, P. Odoi, I. Mpairwe, F. Verity

**Affiliations:** 1 Faculty of Medicine, Health and Life Science, Swansea University, Singleton, SA28PP Swansea, Wales UK; 2 YouBelong Uganda, P.O Box 36510, Kampala, Uganda; 3 Liverpool School of Tropical Medicine, Pembroke Pl, L3 5QA Liverpool, United Kingdom; 4 Walimu, Plot 5-7, Coral Crescent, Kololo, P.O. Box 9924, Kampala, Uganda; 5 College of Health Sciences, Makerere University, Kampala, Uganda

**Keywords:** Severe mental illness, Uganda, Hospital discharge, Family care, Community care

## Abstract

In Uganda, low resources for mental health provision combine with disadvantage and inadequate supports for family and community-based care. Catalysed by the need to reduce overcrowded psychiatric hospital wards and frequent readmissions at Butabika National Referral Mental Hospital (BNRMH) in Kampala, the nongovernment organisation YouBelong Uganda (YBU) developed the YouBelong Home (YBH) intervention. YBH is a theoretically eclectic pre and post hospital discharge intervention. This paper reports on qualitative findings of the project Curtailing Hospital Readmissions for Patients with Severe Mental Illness in Africa (CHaRISMA), which explored how to refine the YBH intervention. The project was funded by a UK Joint Global Health Trials (JGHT) Development Grant. Data was collected through structured interviews with service users and caregivers, reflective practice by the YBH implementing team and a stakeholder focus group. A summary of refinements to the YBH intervention follows the TIDieR format (Template for Intervention Description and Replication).

## Introduction

Response to severe mental illness (SMI) service provision is an ongoing challenge in Uganda (Kisa et al., 2016; Molodynski, Cusack & Nixon, 2017, p.98; Kagaari, 2021). Like other sub–Saharan African countries, the burden of SMI in the country, set against low resources for mental health provision, hampers adequate response from the formal health system ( Molodynski et al., 2017; Kagaari, 2021). Molodynski et al. (2017, p.98–99) highlight other complex factors in the mix; the effects of stigma about mental illness, entwinement of mental illness and poverty, perspectives on evidence-based treatment and an acceptance of pharmacological and psychosocial interventions. This complexity has implications for the health and well-being of individuals living with SMI, their families, community carers, and how best to implement sustainable community-based mental health interventions (Cappo, Mutamba & Verity, 2020).

Although discharge from a psychiatric hospital can offer hope and optimism for a person recovering from a SMI, severe limitations in the availability of community-based care can instead contribute to negative outcomes such as homelessness, suicide, family breakdown, relapse and recurring hospital readmissions (Molodynski et al., 2017; Tyler, Wright, & Waring, 2019; Cappo et al., 2020). Guidelines for optimal hospital/community care transitions (NICE, 2016) though available, are problematic to implement in Low-Income Countries (LICs) (Cappo et al., 2020). Even with a decentralised health system which is an asset to leverage, there is great under-resource in personnel trained in mental health, psychosocial support and an adequate medication supply (Kisa et al., 2016; Molodynski et al., 2017). Furthermore, complex pressures on family carers impact the care transitions (Verity, Turiho, Mutamba & Cappo, 2021).

A systematic review by Tyler et al. (2019) of post-psychiatric hospital support for people living with SMI emphasises the importance of access to medicine, peer support, psychiatric or general practitioner follow up, psychoeducation and psychosocial support. Moreover, stable housing, adequate income and continuity of family care and support are crucial to respond to the risk factors discussed above (Tyler, et al., 2019; Sfetcu, Musat & Haaramo et al., 2017; Kalseth, Lassemo, Wahlbeck et al., 2016; Rosenfarb et al., 2006). Discharge models positively associated with reduced rates of psychiatric hospital readmission have some shared elements. Tyler et al. (2019, p.21) found these interventions link hospital care and care in the ‘community’, utilise relational/person-centred approaches and are based in social health models of care. However, there is a paucity of LIC based research about what works to support effective care transitions for people living with SMI as they return to their homes and families (Tyler et al., 2019).

YouBelong Uganda (YBU) a non-government organization, has aimed to address these complex issues through the YouBelong Home (YBH) intervention. This intervention was instigated in Uganda and catalysed by the aim of reducing overcrowded psychiatric hospital wards and frequent readmissions at Butabika National Referral Mental Hospital (BNRMH) in Kampala, Uganda. Hospital overcrowding and human rights issues have been the subject of community action and public scrutiny (Molodynski et al., 2017).

YBH seamlessly integrates mental health care system transitions, supports self-care and recovery and bolsters family carer support. It combines four practice approaches: (1) relationship-centred practice; (2) culturally centred practice; (3) family-focused recovery and (4) assertive community mental health work. It is delivered by a trans-disciplinary team (social workers, occupational therapists and mental health nurses) working both within the hospital and community setting and supervised by experts in the disciplines of psychiatry, public health, social work and health systems development.

YBH responds to the needs of people living with various mental illnesses including bipolar affective disorder, schizophrenia and other forms of chronic psychosis, epilepsy, HIV-induced psychosis, severe depression, alcohol and substance use disorders. In Uganda, epilepsy is categorized as a neurological condition, but it is also serviced within the mental health system because of the associated stigma and often has comorbid mental health conditions. Many service users live with both SMI and high rates of alcohol and substance abuse yet there are limited options for drug and alcohol supports for people who also experience SMI (Degenhardt, 2018).

The intervention is theoretically eclectic. From philosophy and sociology, it is informed by understandings of the inter-subjective and socially shaped formation of relationships, social connections, capabilities and belonging. YBH also draws on empowerment-based social work, with roots in structural understandings of power relations and change, and family social work, with a basis in ecological systems theory (Gitterman & Germain, 1976). Additionally, YBH uses the assertive community mental outreach approach and evidence-based tools from psychoeducation and family communication (Cappo, et al., 2020). Whilst anecdotally YBH has been received positively by service users, family carers and BNRMH, there is a need for more evidence about its effectiveness.

With the objective of developing a stronger evidence base for the intervention, YBU joined with researchers from; BNRMH (Kampala); Walimu, a Ugandan-registered NGO (Kampala, Uganda) that empowers health workers to address local health problems for patients with severe mental illness; and three universities [Makerere University (Uganda), Swansea University (UK) and Liverpool School of Tropical Medicine (UK)].The study entitled *Curtailing Hospital readmissions for patients with Severe Mental Illness in Africa* (CHaRISMA) was conceived to comprise 3 components: (i) a qualitative study to inform refinement of the YBH intervention; (ii) a prospective cohort study to identify the variables and risks associated with hospital readmission and (iii) a Delphi study to examine other community-based options that could support the implementation of community-based mental health intervention. CHaRISMA was funded by a UK Joint Global Health Trials (JGHT) Development Grant. This paper focuses on the findings of the qualitative study.

The YBH team operate in a consultative and dialogical manner, and this approach embodied the ethos undertaken throughout the study. Except for Swansea based researchers, the rest of the research team lives in Uganda. They include Ugandan psychiatrists, social workers, mental health nurses, and occupational therapists and a public health physician. The Swansea based Principal Investigator (PI) led on the action research facilitation for the Project. A non-Ugandan with a community development background, she was highly conscious of her own limitations in understanding, cultural interpretations and the importance of working collaboratively.

## Methods

The primary question driving the CHaRISMA qualitative study was: *What refinements to a community-based mental health support package (i.e., the YBH intervention) can be made through: (a) iterative field testing at the mental health hospital and within the community and (b) stakeholder engagement?* The methodological approach is broadly informed by understandings of critical realism, defined by Bhaskar and summarised by Fletcher as an ontological approach which views ‘reality’ as constituted by three levels (a) the 'empirical domain of experiences’, (b) ‘actual events’ and experiences themselves and (c) subterraneous ‘causal mechanisms’ behind events and experiences (Fletcher, 2017, p.183). Three methods were employed. Firstly, we conducted semi-structured interviews and focus group discussions involving YBH service users and family carers about their experiences and views on the YBH intervention, including what and how the intervention could be improved. Secondly, we carried out action research involving the YBH team. Thirdly, data from the action research, interviews and focus groups were discussed in a stakeholders’ focus group (held in Kampala in October 2019) comprising people who worked within the Uganda mental health system including a psychiatrist, psychologist, and nursing staff.

### Interviews and Focus Groups

Twenty service users of the YBH intervention and twenty family carers were recruited to be part of semi-structured interviews. These were conducted with a predetermined interview schedule informed by the YBH aims of supporting the movement of people from hospital to home. Interviewees were asked to describe their ‘You Belong Home’ journeys. Service users were asked to describe support they experienced and received during the transition from hospital to home and to answer, ‘What has helped you settle in at home?'. ‘Family members were asked to describe the support they experienced and received that assisted them in care relationships and tasks and to answer, ‘What has helped you in supporting your family member settle in at home?’. Formal written and signed consent was obtained for the interviews. The interviews were conducted at a community church located in Makindye Division, Kampala District. Two focus groups were held with six YBH service users and six family carers from Kampala and Wakiso districts. The group discussions were focused on participants’ experiences of the YBH intervention and what they felt worked, why and what could be improved. Both the interviews and focus groups were facilitated by an occupational therapist and a social worker from YBH team.

An overview of the gender, age and mental illness experienced by YBH service user respondents is shown in Table 1. Service users ranged in age from 19 to 69 years. More male YBH service users took part in the study. In contrast, the study included a larger number of female family carers (92% of respondents).

**Table 1:** Characteristics of YBH service user focus group and interview participants

Characteristic	Focus group participants (6 people)	Service users interviewed (20 people)	Total (26 people)
*Gender*			
Male	4	15	19
Female	2	5	7
*Age*			
18–24	1	2	3
25–34	3	4	7
35–44	-	7	7
45–54	2	6	8
55–64	**-**	-	-
64 above	**-**	1	1
*Illness diagnosis*			
Bipolar Affective Disorder	3	6	9
Alcohol and Substance Use disorders	-	10	10
Major Depression	-	3	3
Chronic Psychosis	3	3	6

The discussions were audio recorded, transcribed verbatim and all names were removed to ensure anonymity. The audio recordings were in Luganda, the local language, and were translated to English by the YBH team, all fluent in both Luganda and English. Data of/from the interviews and the focus groups were entered into the NVivo programme. Reflexive thematic analysis was used to analyse data, a method which foregrounds what Clarke and Braun call '...research subjectivity as a resource’ (2018, p.107). Analysis was a recursive process moving between thinking about the qualitative data using the pre-existing theoretical underpinnings and researcher interpretations of what respondents had said to the interviewers. This process was conducted by the PI and Ugandan co-investigators. During this process, convergent and divergent themes and patterns in people’s stories were identified (Clarke and Braun, 2018). Discussion resulted in refinement of emergent themes.

### Action Research

The Ugandan-based YBH team participated in action research to intensively explore YBH and what worked and how it could be improved. In preparation for the action research, a day-long training session on action research was conducted in Uganda, facilitated by the study PI. Nine participants representing three disciplines (social work, occupational therapy, and mental health nursing) underwent two iterative cycles of action research that used individual- and group-based ‘reflection on action’ (Schon, 1983) to draw out characteristics of the YBH intervention that worked and where improvements could be made. The individual reflection on action concentrated on activities within a two-week window, whereas the group-based reflections were done at the end of each cycle.

During both action research cycles, the YBH team completed action/practice diaries. Each diary entry began with a brief, anonymised case description to give some context for the individual-based reflection. Team members then reflected on their engagement with one service user in the previous fortnight to think about what worked well, why, and how YBH could be improved. The study PI read all practice diaries and extracted common themes which were then shared back to the group as a stimulus for group discussion. As part of this process of theme generating, before the first cycle group discussion, she met individually with each YBH team member to discuss their interpretations of the diary entries. Reflective discussions, held in the YBU office in Kampala, were structured by existing Standard Operating Procedures (SOPs) and theoretical perspectives used in the YBH intervention and the emergent themes. The discussions focused on identifying problems, ambiguities, or inconsistencies of YBH and highlighting areas that could be changed or modified. The changes and insights from the workshops were written up and reported back to the YBH team as the final process in action research cycles.

### Focus Group of Stakeholders

To widen the views on the YBH intervention and sense check the findings, a two-hour focus group was conducted with 4 mental health stakeholders and facilitated by a psychiatrist, in their capacity of the Head of the Department of Psychiatry at Makerere University. To stimulate the discussion, an overview of the main themes emerging from the data collection was provided. This was a synthesis of the perspectives from service users and carers and the insights of the YBH team through the process of action research. Analysis was a recursive process undertaken by the external researcher and one of the Ugandan based researchers. Ethics approval for the CHaRISMA qualitative study was obtained from Swansea University College of Health and Human Sciences Ethics Committee, Makerere University School of Biomedical Sciences Institutional Review Board and Uganda National Council of Science and Technology. Relevant permissions were obtained from BNRMH.

## Results

We provide an overview of the lessons for the YBH intervention drawn from the key findings from service user and family carer data, the reflective process with the YBH team and stakeholder focus group.

### Perspectives from Services Users and Their Family Carers

Two sets of themes emerged from these data. The first set is about transition journeys from hospital to home with five themes: ‘*meeting basic needs’*, ‘*drugs and alcohol’*, ‘*being with my people’*, ‘*family support’* and ‘*medication*’. The second set emerged from analysis about what has worked and what could be improved in the YBH intervention. These themes are ‘*relational engagement’*, ‘*focus on recovery’*, ‘*counselling and education’*, ‘*care and regard’*, ‘*transport*’, ‘*referrals to other services’*, ‘*livelihoods supports’*, ‘*family support’*.

An expressed view in YBH service users’ and carers’ stories was the positivity of connecting to family in homecoming, in the words of one respondent of ‘*being with my people’*. For one family caregiver, the experience of having their relative home contrasted with how they saw their relative in hospital *‘….in the hospital you would find her depressed and lonely’*. The data highlight the importance of relationships: ‘*having family around’*, ‘*talking together’*, ‘*staying in touch’*, ‘*rebuilding relationships’*, ‘*reuniting with family’*, ‘*having someone that cared’* and having a ‘*sense of hope’*. These links are a potential source of love and hope and a counter to the pain of rejection and abandonment associated with culturally based stigma of mental illness in Uganda, as elsewhere. These links are also described as having restorative powers, in the use of language such as ‘*rebuilding’* and ‘*strengtheni*ng’.

The link between family care and service users feeling ‘better’ was apparent in several interviews. A closer examination of the interview transcripts highlights some aspects of the care relationship viewed as important. The first respondent communicates care support as having family around, talking together, and staying in touch. Talking and communication is a theme in the following interview extracts from family caregivers, showing the importance of contact and connection.

*My family has been supportive, and I feel much better having them around me. We talk and keep in touch and also taken good care of me (sic).* (Service User)

*Having time to talk to him and keeping the communication which helps me know whenever he has any problem.* (Family Carer)

*We bought him a phone to keep in touch with his family and friends as well which has made him happy. His friends come to visit him a lot which I think gives him hope to know that people out there still care about him.* (Family Carer)

‘Rebuilding relationships’, ‘reuniting with family’, ‘having someone that cared’ and having a ‘sense of hope’ are discussed by the following respondents, 2 family care givers and a service user. The first extract from a family caregiver noting the hope inducing role the YBH programme played in reconnecting the service user with their children. Hope is named in the other extracts, the last one from a service user.

*Keeping in touch with her family especially the children who had abandoned her. It really gave her hope after the YouBelong reunited her with the children. I have also observed that the period she has taken without relapsing is longer than we have ever expected. We are really grateful because we have also been strengthened that she can keep longer at home.* (Family Carer)

*Being there for her because she was rejected by her other family members. I believe that gave her hope knowing there is someone caring for her.* (Family Carer)

*They also helped to rebuild the relationship with my family. They actually gave me hope to recovering quickly. Their advice helped me a lot.* (Service User)

The following extract from a family carer, illuminates the multiple components of post discharge support provided in the context of family care; communication and talking, feeling loved, support to get basic needs met such as clothing, housing and income, and having a phone to stay in touch with family and friends.

*Talking to him as a family has helped him a lot. The siblings also provide him with some needs like clothes which has helped him feel loved. I managed to get him a small house so that he can be accommodated and also encouraging him in small family business like poultry. I also bought him a phone to help him keep in touch with us especially when he moves away from home to visit his friends.* (Family Carer)

In a focus group a service user talks about YBH intervention supporting them from being ‘stuck’ in hospital to having a way to ‘get out’ of hospital. They note the visits and discussions in both the pre- and post- hospital stages.

*…before YouBelong came to me, I was stuck in the hospital without any way to get out. When they came, they created a way for me to get out of the hospital. They keep having discussions with us and our families until they are able to take us home. They visit three times after taking you home; encouraging you and guiding you on how to take the medication.* (Service User)

Caregivers affirmed the recovery focus of YBH. The cultural understanding of mental illness is seen in a static context, whereas the recovery concept is dynamic and evolving. Respondents noted the value of counselling and education to the individual and the family about mental illness, medication management, achievement of personal goals and ways to deal with stigma; for family members, this shift underscored approaches to enhance the care they provide. The following responses demonstrate these themes:

“*I emphasize the visits were very helpful and we were educated on how to take care of x. He now takes his own medication and the phone calls were also useful*”. (Family carer)

“*They have always come and discussed with us a number of questions regarding the client. Before they talked to me, I had a lot of hatred towards the client following the manner how he used to mistreat us (following getting drunk from alcohol) but later they helped me calm down. They offered us counselling and took good care of us. They really calmed us down from the hatred we had towards the patient. I think the patient was also counselled while in the hospital because for the first time he was so calm on his return as opposed to the previous times when he would still be harsh on the family*”. (Family carer)

An important component for recovery is access to medication (Cappo et al., 2020). Within the interview data, access to medication is linked with access to health clinics for prescribed medication refills, transport access and access to funds. Respondents also commented on the need to have adequate knowledge about the function of medication and how and when they are to be taken (i.e., not with alcohol). Service users and family members pointed out the need to have medications that do not have visible and disabling side effects, like trembling hands. The YBH team provided transport in the YBH bus to take people to their homes and communities at time of discharge from hospital. There were many positive comments about the appreciation of this transportation, and the ‘*eats*’ that are provided on the bus. Ability to access transportation is a major financial challenge or often beyond the reach of many people in LICs such as Uganda.

“*The family has been able to buy him medicines in time especially when he gets less supply from the hospital. He has also been provided with transport to go back for review and medicine refill in the hospital. The medication that they provided the patient were very helpful*”. (Family carer)

“*They have advised me to take my medication and their advice has really helped me. It has now taken me about one year without relapse. They have also helped me bring my medication especially making sure I get the one that works for me*”. (Service User)

In LICs such as Uganda, transport infrastructure is poor. Roads are in a poor state, and in places difficult to access, even within 30 kms of Kampala (the capital city of Uganda), which is the current YBH catchment area. Alongside the practical movement of a person to home, the YBH transport seems to have other symbolic attributes evoking a spirit of care, support and a positive process of embarking on a new journey.

The role of the family in meeting basic needs (shelter, food, livelihood support, love) is a thread throughout the interview data and reinforces how foundational these needs are to being settled at home. Lack of an income or the means to earn a livelihood was named as a stressor for some participants. Some respondents had their education curtailed because of their mental health issues. These matters raise questions about how to support ongoing opportunities for social participation, education and income generating activities (IGAs) in the YBH intervention and develop various stages of such activity for service users right for their situations.

*“The supports are only effective with adequate family engagement and community support to follow up on some issues in our absence. Increased participation and engagement is affected by perception of family members and community members towards mental illness. Most times the families are burdened with care for their family member and also bear the stigma”. (YBH team reflection)*

The YBH intervention is based on an engagement model which includes visits to patients on the convalescent wards of the mental health hospital, to make introductions and to undertake the comprehensive assessment and home visits to family and the service user once they go home. This model is valued by the interviewees, as seen in the extracts below.

*“I liked the visits a lot and would be very happy knowing that they are going to visit”.* (Service User)

*“They approached me in the hospital and gave me time to talk to me. (sic) I actually appreciated the long time they gave me to talk to me. It’s rare to get someone at the hospital to talk to you and listen to your concerns and I felt so good when they came to talk to me”.* (Service User)

*“They encouraged me take care of myself and now am presentable in the community and also go to church to pray. I was always happy each time I would know they are going to visit me because I would learn a lot from them”.* (Service User)

### Perspectives from YBH Team Reflections

The team’s reflective diaries and team discussions raised the complexities of the YBH work, and in different realms, including the context of the issues facing service users and families and the low level of social protection supports. Service users’ range in age (i.e., from 20 years to 70 years) and include both men and women. Repeat hospital admissions were common in the diary accounts. In the YBH post-discharge work, service users are discharged to reside with ‘family’ members, however they define them. It may be those with whom they have kin relationships, a bond or connection and capacity to be in a caring role. Rejection, abandonment, family violence, family conflict and challenges and feelings of ambivalence on the part of family caregivers are recurring themes. It is common that people are not visited by family whilst in hospital. For example, in one diary it was noted: *“She has never been visited by any of her relatives in her current readmission which is now over two months”.*

There were examples where, due to past experiences usually associated with violence or community stigma, families are reluctant to have people return home following an episode of hospitalisation. The YBH team could face a major challenge in finding a home for the service user to return, and relatedly, in building both the service user’s and family or caregiver’s confidence and faith for this transition to happen. These factors are critical to understand during the pre-discharge process. The human rights issues for people with lived experience of SMI were evident, for example, the right to a safe environment, to be accepted with equal dignity and to effective treatment.

Although the mission of YBU is to support people to leave hospital and return home, reduce unnecessary hospital admissions and support individuals and families in processes of mental health recovery, the YBH team discussed how some service users expressed discomfort with or apprehension about returning to their family home or to live with extended family. One diary entry notes that a service user “…*believes he was left in hospital by his family as a punishment”.* In another case, the YBH worker records in a diary entry that the service user “*…had escaped from her family that she claimed attempted to kill her three years ago*” and “*She also has fears of going back home for (sic.) and rejection”.* In another case, the worker records in a diary entry that a service user “…*was not willing to go back home because she was always locked in the house and was not given freedom to get involved in other community activities*”.

From the perspective of YBH team members, in the main, organisational elements of the YBH intervention were seen to work well. These elements included the structured format and standard operating procedures, the trans-disciplinary team, team training and culture of learning for improvement. Shared values, cooperation and respect for culture and belief in mental health recovery were identified as important in the YBH delivery. Also pivotal are the practical mechanisms for YBU’s engagement and collaboration within the BNRMH wards. Reflection on action illuminated how YBH existing protocols could be fine-tuned. Improvements were identified in the conduct of YBH case management and in how the YBH team works with the hospital (i.e., alignment of hospital assessments and YBH assessments and links to hospital physical health records). There was also discussion on how to continually deepen ‘empowerment’ practice in the context of working co-productively with people living with SMI in Uganda. Outside these improvements, there was discussion about providing more family conducive spaces within BNRMH so that families were comfortable in visiting their family member and had privacy in the pre-discharge processes.

The YBH team reflected that there were areas where the pre-and post-discharge work could be strengthened. The YBH team engage with the service user on the hospital ward and time is taken to understand their circumstances and personal goals. This process takes time and needs to be conducted when the service user is best able to talk about themselves and their goals after discharge from hospital; prescribed treatment during their acute presentation, however, might impede this type of discussion. It also was important that time is taken to develop a relationship with the YBH worker. There was also room to improve the pre-discharge mapping tools which had evolved to be more complex than need be and reconsider the purpose and administration of the self-esteem and stigma tools. These tools presume a level of literacy by the service user.

A strength in the post-discharge work was the empowerment plan developed in the pre-discharge phase and the creative delivery of both psychoeducation and support for community links and social skill development. With respect to improvements, these focused on need for greater clarity of the role of YBH in income generating activities (IGAs), stronger support for service users to participate in activities once home and social support development (i.e., links to village-based communal activities).

### Perspectives from Stakeholders

Overall, the YBH intervention was viewed by stakeholders as a positive initiative. As a stakeholder said:

“*I think that this is a very, very nice study and I think it is addressing a very important need in this country. The idea that a patient can no longer return home or is brought and no-one is going to follow him up has been a problem for us for a long time. I think YBH is coming into filling a very big gap*”. (Stakeholder Focus Group)

There was endorsement of a trans-disciplinary team, the values of YBH and the broad range of strategies used in the pre- and post-discharge phases. Stakeholders recommended that the intervention is written into a manual and protocol and that there is staff training to ensure that intervention components are delivered with required expertise. On the latter point, there was debate about whether the YBH delivery team are best generalists in community mental health or if they should be specialists with expertise in, for example, psychoeducation or family communication skills. The stakeholder group suggested that peer workers and peer supports is an area where YBH could expand and develop.

There was a lengthy discussion in the focus group about the need to address the manifestations of family stigma about mental illness and the implications for care and support. As one stakeholder said:

“*I think the education is doing its job, educating the carer goes a long way, but we are stuck on the stigma for the family, because if I have mental illness I am stigmatised but so is my family. They may be ambiguous, they want to support me, but the stigma is getting in the way. So, for me, I think I’ll still go back on that, that if we can get a way through education you’re working with that, but one of the major barriers/factors to mental health care is that the care giver/carer feel as stigmatised as the patients, sometimes even more*”. (Stakeholder Focus Group)

From this discussion there was a view that an intervention like YBH needed to support families in dealing with stigma, including addressing stigma internalised by children within the family. A related suggestion was to include a stigma assessment tool for the family member or caregiver as part of the YBH intervention. In the current model, the tool is used only for the service user. As another respondent said:

“*I am just wondering if we had stigma tools and suggestion tools for the caregiver, whether that wouldn’t give us more information on recovery and everything, because I think… my personal experience is that how caregivers feel about a patient, their relative and the stigma around the patient does not only impact on the patient, it also impacts on the caregivers and therefore that can impact on the duration of the time before they come back to the hospital, because the caregiver feels really stigmatised about mental illness. The chances are that the stigma is going to negatively impact the care they give*”. (Stakeholder Focus Group)

Sustainability and effectiveness of YBH in the context of high needs and limited resources was a concern. There was a perspective that the current model, with YBH being based near BNRMH and outreaching to service users and families in the post-discharge phase, would eventually be, in the words of one stakeholder, ‘overstretched’. An alternate model is for YBH to comprise small teams placed in local health centres with local knowledge. As a respondent said, *“So I think that if we got somebody, even if not an entire team there to operate within the community, within close proximity, somebody who understands that general area, that might help”.* Relatedly, there was a view that connecting as much as possible with local community leaders and other community outreach workers and development projects was important to sustain the value of the YBH intervention for service users and families. This is in keeping with the current format of the post discharge empowerment intervention.

The YBH team must manage limited resources and high levels of need. The dilemmas that arise permeate all aspects of the intervention and require the management of service delivery expectations. Planning for the end of the intervention, and the parallel process of consolidating supports, is vitally important and requires clear structuring throughout each step of the YBH post-discharge work. This is particularly so for the phone contact which is the final stage of the YBH intervention. As one stakeholder said “*…there’s structure with the timing of the phone calls, but there’s also structure on the questions to be asked”.* Related was a view that YBH needed to stay in ‘scope’, given the extent of needs and in the words of a stakeholder, to not take “…*on much more than it can actually take on”.*

In summary, the CHaRISMA qualitative research explored multiple perspectives on what is working well in the YBH intervention and why, and where there was scope for improvement. Subsequently, the YBH team have refined YBH and deepened a collective understanding of what is working well. CHaRISMA has given confidence that the YBH intervention is appropriate and acceptable in the Ugandan context. Through the period of the CHARISMA study and subsequently, work has been done to improve community development processes and links to community leaders; enhance supports for reduction in the use of substances (drugs and alcohol); address mental illness stigma in families and expressed by children and develop ways to improve the use of phone supports.

Moreover, through CHaRISMA the YBH team has increased research mindedness, and a culture of enquiry and improvement. This has continued beyond the life of this research and was to be significant in how YBH adapted during the COVID-19 pandemic. The YBH team have observed that engagement in CHaRISMA guided them on important skills that team members need to develop to enable them empower people recovering from mental illness and their families. Refinements to the YBH intervention and a summary of areas in YBH supported by the research evidence are shown in Table 2, which follows the TIDieR format (Template for Intervention Description and Replication) (Hoffmann, Glasziou & Boutron et al, 2014).

**Table 2:** YouBelong Home using the TIDier format (Template for Intervention Description and Replication)

TIDier format Item	YBH Intervention Description using the TIDier format	YBH area endorsed by CHaRISMA study	YBH area refined because of CHaRISMA study
**Brief name**	YouBelong Home (YBH), a 16-week pre- and post-discharge reintegrating people hospitalised with Severe Mental Illness (SMI) back into their families and local communities.		*Refined* The intervention has increased from 14 weeks to 16 weeks
**Why**	YBH is informed by theoretical understandings on belonging, mental health recovery, culture, family care and human rights. These are reflected in YBH values and principles. These are used in a model of practice that combines relationship-centred practice, culturally centred practice, family-focused mental health recovery and assertive community mental health. 1. *Personal identity* formed in social relationships. 2. *Belonging* to family, community and culture, is a basic human need and pivotal for mental health. 3. *Recovery from SMI* is an active participatory process led by the person in recovery. 4. *Community-based mental health services* should be the centre piece of mental health care, providing the person in community with ease of access to care ‘in community’. 5. *Provision of community-based services and supports*, including innovative practices, should only be implemented if they are of high quality, scalable for significant impact in the community and sustainable. 6. *The family unit*, the* local community* from which a person recovering from SMI comes, and *tribal* culture (in Uganda and other African countries) are essential resources in the recovery process. 7. *Building opportunities and skills for families*, particularly of the main carer in the family empowers families and strengthens their capacity to respond to the range of issues associated with having a family member recovering from SMI. 8. *Cultural context, awareness and sensitivity* should be the core driver in education of families and communities in mental health. 9. *The decentralized health system* in Uganda is a key resource. 10. *Human rights* values and principles.	The focus on recovery, belonging, family and relationship centred and culturally centred practice was affirmed.	An explicit human rights values principle was added.
**What**: Materials	**Memorandum of Understanding (MOU)**: YBU has a formalised written MOU with BNRMH, stating YBH team’s role and functions and collaborative arrangements. YBH has 5 Standard Operating Procedures: ‘Introductions’, ‘Income Generating Activities’, ‘Medication adherence’, ‘Dealing with challenging behaviours’, ‘Conflict resolution’ and ‘Crisis management’. **Pre-discharge**: The predischarge assessment phase is a co-production process utilising 5 tools. i. General health assessment and mental health assessment (with service user) ii. The Rosenberg Self Esteem Scale Tool (with service user) iii. The Internalized Stigma of Mental Illness Inventory-9 item Version (with service user) iv. Mapping of resources and assets in the service user’s family and network of social relationships (service user and the family carer) v. Co-produced personal empowerment plan for post discharge (outcome of assessments) **Post discharge**: Material used in this phase are mental health education resources packaged in terms using local languages that are cultural and belief sensitive.	Importance of the MOU with the hospital and close relationships with the nursing staff confirmed. A dialogical process of assessment and communication based in empowerment values.	Improvements were made to the interface between YBH and hospital ward processes. The pre-discharge assets and resource maps were simplified to ensure they capture what is necessary for an empowerment focused assessment. Refinements to the following Standard Operating Procedures: ‘Medication adherence’, ‘Crisis management’, ‘Conflict resolution’, ‘Income Generating Activities’ and ‘Dealing with challenging behaviours’ Arrangements were made with the hospital for the YBH team to write in case notes. Mental health education resources were changed using local languages that are culturally and belief sensitive.
**What: Procedures**	**Predischarge assessment phase**: Co-production meetings with service users in the hospital convalescence ward to undertake assessment and co-produce an empowerment plan. Meetings (x2) are held with a family member, including one in the home setting. YBH transports service users’ home in the YBH bus with celebration of home coming with food. **Post discharge activities**: Through home visits and phone support, strategies that might be employed are mental health psychoeducation, medicine access, family relationship and personal development counselling, access to livelihoods support, links to community leaders and addressing stigma and discrimination. Termination and referral of the YBH service user to the nearest health centre for medication refills. **Continuous quality improvement**: Through reflective processes, shared working and case management, evaluation and research culture, and feedback loops between YBH and the hospital. YBH staff have access to hospital wards and meet with key hospital staff.		The pre-discharge phase was extended from 2 to 4 weeks to strengthen the preparation of the service user for returning home. More attention on psychoeducation about a service user’s diagnosis and treatment and on motivational interviewing for service users recovering from substance use disorders. Additional hospital visit by family member prior to discharge to support family understanding of care needs. Modifications in the post-discharge phase. Increased focus on IGAs and tightening of the structure of phone support practices. Team working processes and critical reflective practices strengthened.
**Who**	• YBH is delivered by employed trans-disciplinary staff from disciplines of social work, occupational therapy and mental health nursing. • Staff have community mental health knowledge, communication skills, team working skills, cultural understandings and personal qualities and values (i.e., empathy, compassion, belief in human rights, empowerment and change). • There is mental health and social work leadership. • Staff are engaged in regular training and development about aspects of the YBH intervention, such as interviewing, assessment, case management and community development.	Endorsed the configuration of a MD team for the delivery of YBH.	
	The YBH intervention has three modes of delivery through: i) Individual case work ii) Family work iii) Community development. The pre-discharge work in the hospital setting is initiated by YBH workers, subject to the service user consent. It is conducted on a face-to-face basis. The post-discharge work is conducted face-to-face initially and then replaced by telephone calls with individuals and families. The community development work in the post-discharge phase is delivered with individual community leaders, with groups of community leaders or local community members and with staff at district health centres.	Endorsed	
Where	In Uganda, the YBH intervention is delivered in the districts of Kampala and Wakiso. **In the pre-discharge phase**, the intervention occurs in the hospital convalescent ward and includes family home visits. **The post-discharge work** is in the service user’s home environment, which can be a village or urban dwelling. It also takes place in the local community and health centre.		
	The intervention will occur once the person is ready for discharge from the hospital. There are 4 weeks of pre-discharge work, with 2–3 assessment sessions tailored to the circumstances of the service user. Post-discharge activities occur over 12 weeks and comprise 2 blocks, each 6 weeks in length; block 1 comprises two home visits, block 2 comprises 2–3 phone calls followed by one termination visit.		
	All participants receive the same intervention which is personalised and based on needs, family situation and personal empowerment goals. Role plays and case analysis help fidelity.	The use of role plays and case analysis to assist consistency of implementation across the YBH team.	

## Discussion

Developed in Uganda, the YBH intervention provides a structured family and community reintegration process following hospitalisation (Cappo et al., 2020). CHaRISMA findings indicate it is a well-planned model that supports the movement of individuals with lived experience of SMI from hospital to family and community life. This model includes a pre-discharge phase that co-produces a post discharge empowerment plan based on a relational assessment process. This phase unveils factors that are important to the smooth and dignified movement of the person to their family and community, in ways that enhances the person’s belonging and supports their empowerment and health goals. The post-discharge follow-up phase supports the person discharged into active family and community life. Significant barriers to achieving a functional life and enhancing individual capabilities of people recovering from mental illness are addressed through a family- and community-centered approach.

The YBH model is focused on strengthening non-hospital based mental health care in a low-income country as recommended by Thornicroft & Tansela (2003). It aligns with key aspects of WHO’s mhGAP Intervention Guide (mhGAP-IG), namely in its principles (communication, respect, and dignity), the recommended psycho-social and psychoeducation practices and carer supports (mhGAP-IG) (WHO, 2016). Furthermore, there is a parallel between YBH and the characteristics of effective interventions explained in Tyler et al’s (2019) study of post-psychiatric hospital support for people living with SMI. YBH’s focus on family as a resource and strengthening family care relationships and capacity to undertake care tasks, resonates with the theoretical work of Keating, Eales & Funk, et al, (2019, p.149) who conceptualise care as ‘tasks’ and ‘being in relationship’. As evident in our research, dynamic challenges exist in the hospital to family care journey for the person living with severe mental illness and their carers. There is a solid body of knowledge on the significant barriers to community based mental health care and support, coupled with family stresses. An example is the extensive literature on expressed emotion (EE) in family systems towards a person living with SMI (Brown, Carstairs & Topping, 1958; Sfetcu et al., 2017). These stresses and strains are compounded in LICs by a lack of health care infrastructure, human resources for health and the daily impacts of poverty.

Mindful of the need for a holistic response to complex issues, the YBH intervention was purposefully designed to integrate understandings and tools from social work, sociology, public health and mental health. A key underpinning is the notion of belonging (Cappo et al., 2020). Interview and focus group data indicate the importance and value of belonging and human relationships, and YBH’s recovery and empowerment orientation. In the Ugandan context, this recovery focus requires significant team skills in counselling for the change of conceptual thinking to be taken on by service users and families. Fundamentally important in the YBH program, is immersion in the local cultural context. It is central in YBH teamwork, to connect with faith healers or traditional healers, in local villages, with whom the service user or their family have or continue to engage with. While some traditional healers reject any dialogue with Western pharmaceutical intervention, and discourage its use, others are open to further discussion, and at times, cooperation (Ovuga, Bordman, & Oluka, 1999; Semakula, 2020). While the experience of the YBH team would indicate that there are many steps to take before some type of health structure might be developed that combines different beliefs, the CHaRISMA experience has led the YBH to formulate a program of contact, dialogue, cooperation, leading to the possibility of mutual referral.

The YBH team works to bring together transdisciplinary knowledge, the value of this was evident from the team reflections on shared problem solving and case management. There were many cases where the team, working co-productively with people with lived experience and carers, were required to use considerable ingenuity to implement the pre and post discharge supports. Again, this requires a certain holistic thinking beyond the realm of a single disciplinary perspective.

Drug and alcohol issues feature for several people who had used the YBH intervention. Uganda has a high levels of alcohol consumption (Swahn et al., 2020). Our data highlight the importance of attention to supporting people in making changes to cease or reduce the use of substances, and the value of both advice and counselling on drug and alcohol use/behaviour change, and what can be put in place as an alternative. To what extent does the current YBH programme support this work, and how might this be further developed was a question arising from the study. Furthermore, findings shows that access to transport is an essential component of the recovery process after hospital discharge Respondents raise access to income, schooling, and health centres, as components of longer-term support, and this accords with the social determinants of mental health.

The interview material raises questions and dilemmas about processes for more longer-term support when an intervention like YBH ends. Firstly, in an intensive relational programme like YBH, how do ‘endings’ happen in ways that reinforce the supports that have been provided, for example the psychoeducational lessons, and the ‘hope’ which is mentioned in these interviews? Secondly, are there possibilities for referrals to other providers (government and non-government providers) who can pick up the support processes? With Uganda being a LIC, with an under resourced health system, and without a formal community based mental health system, the referral process by YBH team to the local health centre, often, results in no more than medication refills.

One long term strategy that YBU has been engaged in is training of health workers in mental health, so that health centres can become a resource for mental health support. However, in the short term, YBU should consider other strategies of referral, (e.g., peer support, or family to family peer support). The key point is that ongoing support will need to come from within the community, given the limited capacity for formal services to be available for this long-term work. Significantly, there is need to work with the already existing structures in the health system to embed this model into the system, including at different levels of health centres and hospitals that provide health services to the people in the community. Thus, working with them will empower and enable them to provide better mental health services at the community level, including but not limited to training health workers, developing a good supply chain of essential mental health medications to primary health centres, and working with the mainstream health.

There are study limitations. The qualitative interview and focus group data were from a small number of people and may not have reached saturation however, given the diverse situations for people. A more comprehensive picture of the characteristics of the people using the YBH intervention were collected in a parallel but separate CHaRISMA Risk Score Study. The aim of this sub-study was to establish baseline knowledge on the experiences of people living with SMI during their hospitalization and the recovery journey after leaving hospital to inform further refinement of the YBH intervention. A larger evaluation will deepen insights on the impact of the intervention in use and its cost-effectiveness. For this qualitative study, no quantitative information was collected about YBH’s impact on people’s mental health, hospital readmissions and family care and relationships. In addition, the CHaRISMA study lacked direct service user participation on the research team. It is of note, however, that the dialogical nature of the CHaRISMA study has more recently led to the formation of consultative group discussions with young Ugandans in recovery from SMI, and their carers, leading to direct influence on policy and program implementation.

In conclusion, the refined YBH intervention in Uganda has demonstrated an intervention well-received by study respondents that is theoretically informed and grounded in human rights, social justice and mental health recovery principles and integrating care systems and approaches. This study demonstrates that an intervention like YBH, has promise in moving towards community-focused mental health care in low resourced settings. Further research will generate more robust data on their cost-effectiveness in improving health and wellbeing outcomes for people living with severe mental illness and those in their informal care system.
